# Supplementary material for: speedingCARs: accelerating the engineering of CAR T cells by signaling domain shuffling and single-cell sequencing
Source: Nat Commun. 2022 Nov 2;13:6555. doi: 10.1038/s41467-022-34141-8 (PMC9630321; doi:10.1038/s41467-022-34141-8)
Supplement: Supplementary file 1 — Supplementary Information [file 41467_2022_34141_MOESM1_ESM.pdf]

speedingCARs: accelerating the engineering of CAR T cells by signaling domain shuffling and single-cell sequencing

Rocío Castellanos-Rueda*[1,2], Raphaël B. Di Roberto*[1], Florian Bieberich[1,2], Fabrice S. Schlatter[1], Darya Palianina[3], Oanh T. P. Nguyen[1], Edo Kapetanovic[1], Heinz Läubli[3], Andreas Hierlemann[1], Nina Khanna[3] and Sai T. Reddy [#, 1]

[1]Department of Biosystems Science and Engineering, ETH Zürich, 4058 Basel, Switzerland

[2]Life Science Zurich Graduate School, ETH Zürich, University of Zurich, 8057 Zürich, Switzerland

[3]Department of Biomedicine, University of Basel, 4031 Basel, Switzerland

*Co-equal contributors

[#]To whom correspondence should be addressed. Tel: +41 61 387 33 68; Email: sai.reddy@ethz.ch

SUPPLEMENTARY INFORMATION

SUPPLEMENTARY FIGURES 1 – 15

SUPPLEMENTARY TABLES 1 – 3

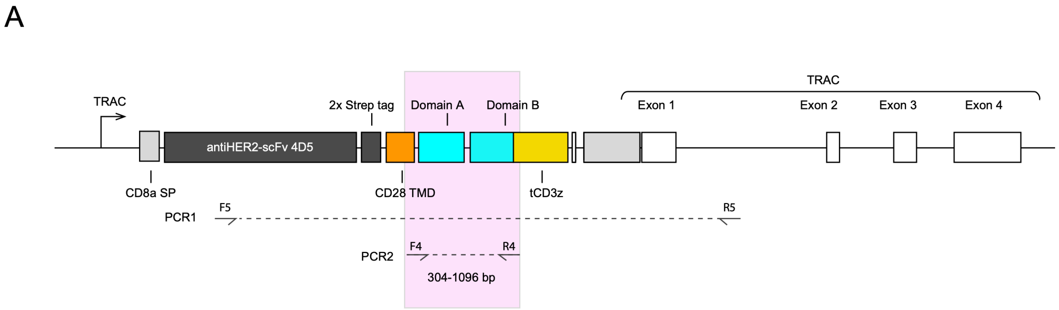

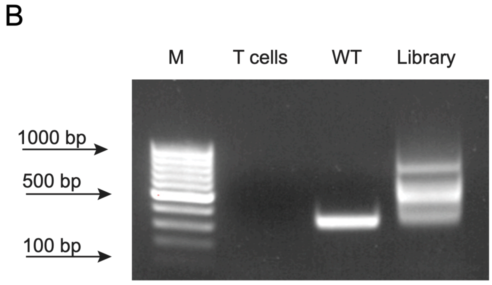

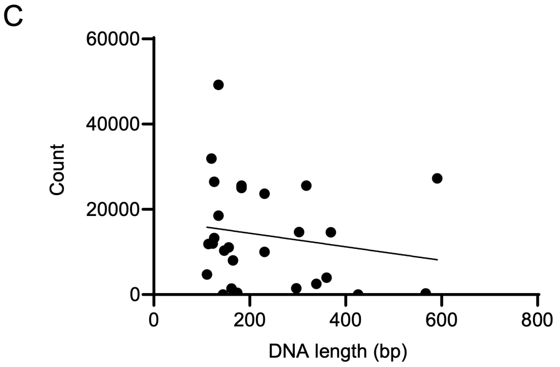

Supplementary Figure 1: PCR amplification confirms the integration of the CAR library.

a Schematic representation of the PCR amplification strategy to obtain amplicons of the integrated CAR gene. **b** Agarose gel electrophoresis of the genomic amplicons from primary T cells (T cell), 28z CAR (WT) and sorted CAR T cells expressing the library of signaling domain variants (Library), all run alongside a DNA molecular weight marker (M). The library lane shows the range of expected amplicons owing to different signaling domain sizes. **c** Scatter plot of the signaling domain's DNA length against their abundance in the CAR T cell library (post transfection, post sort). There is no significant correlation between the two variables.

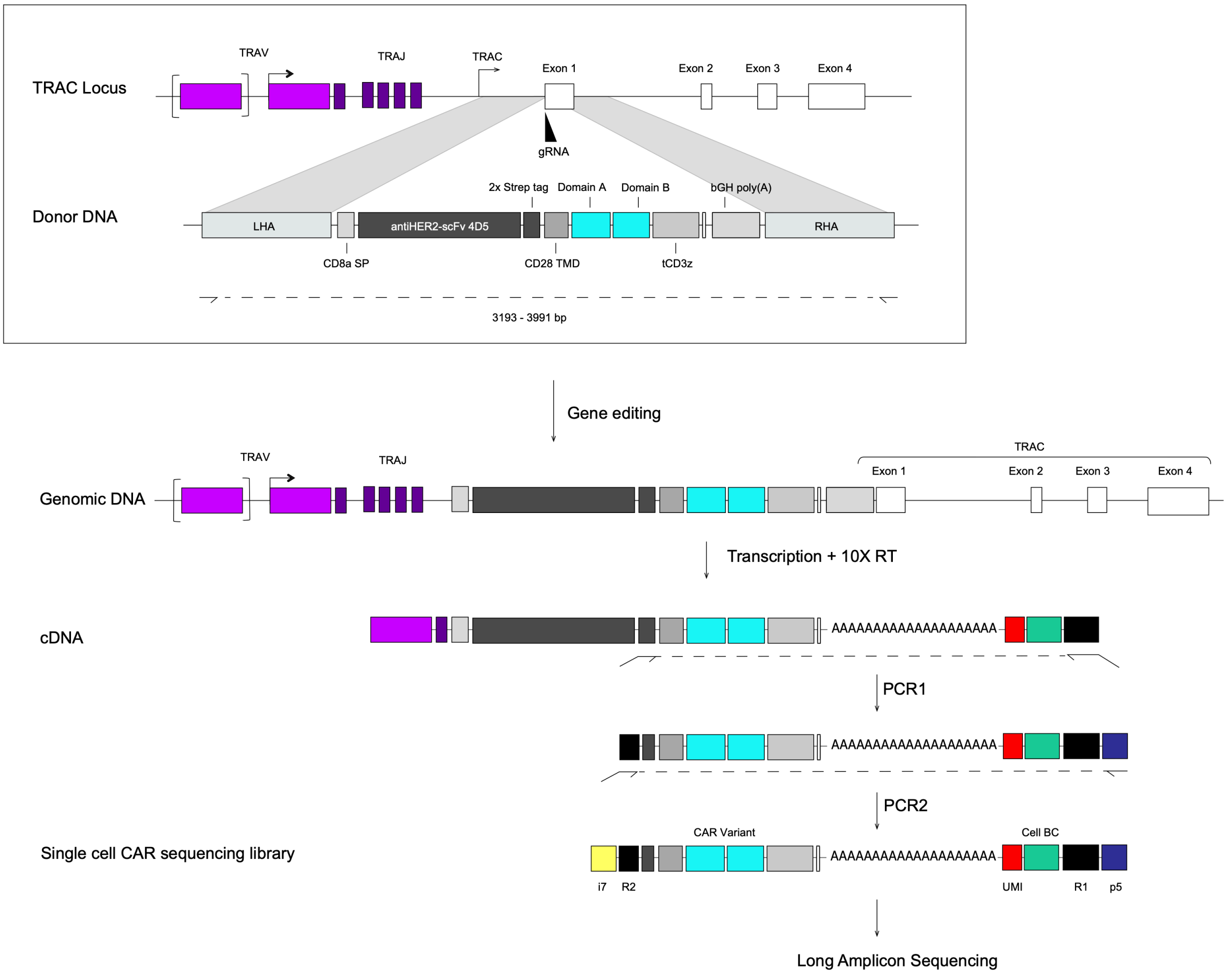

Supplementary Figure 2: scCAR-seq PCR amplification strategy for library de-multiplexing.
Following CRISPR-Cas9 based gene editing CAR T cells incorporate a single copy of a CAR gene into the *TRAC* locus. Guided by *TRAC* specific gene expression regulation the CAR gene is transcribed into mRNA and translated into a CAR. During 10X droplet encapsulation, reverse transcription and cDNA synthesis each CAR transcript incorporates a barcode specific to its cell of origin (cell-BC). A two-step PCR amplification strategy that makes use of the synthetic Strep tag sequence found in the CAR gene, allows to selectively amplify cell-BC linked CAR transcripts from the 10X cDNA mix. This scCAR-seq library can then be sequenced using long amplicon technologies (PacBio) to trace the origin of CAR transcripts to each individual cell identified in the 10X gene expression pipeline.

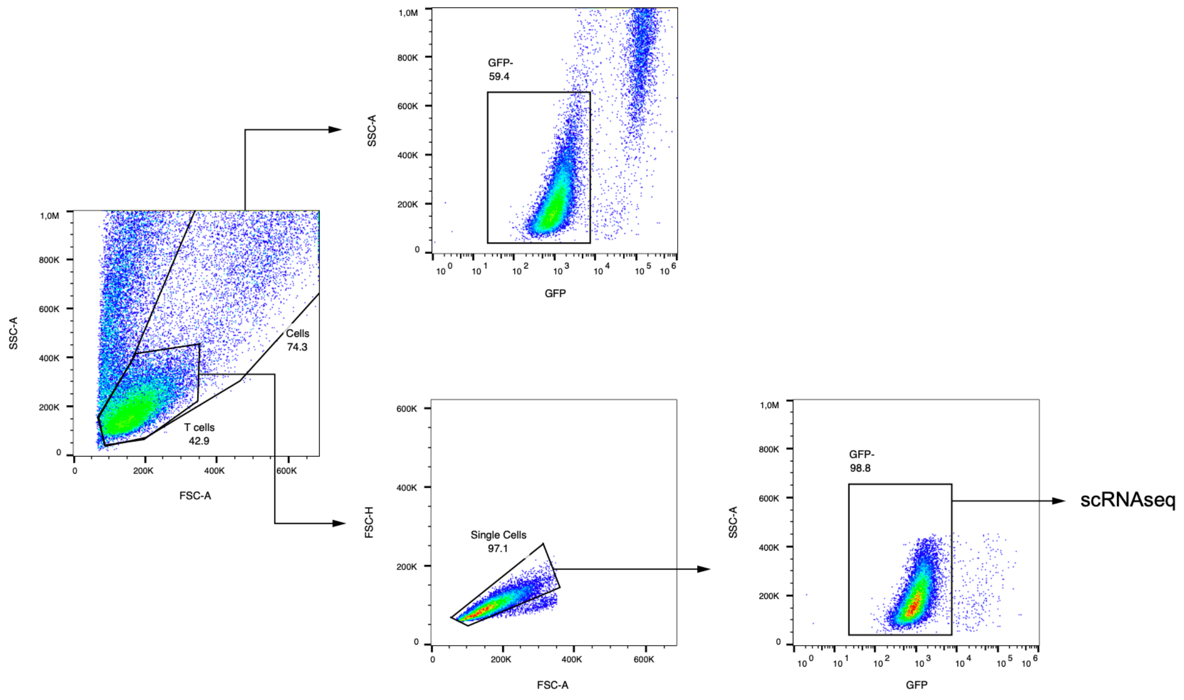

Supplementary Figure 3: FACs gating strategy used to isolate T cells following tumor co-culture.

Following 36h of co-culture between CAR T cells and SKBR3-GFP tumor cell lines T cells were sorted based on size, density and lack of expression of GFP.

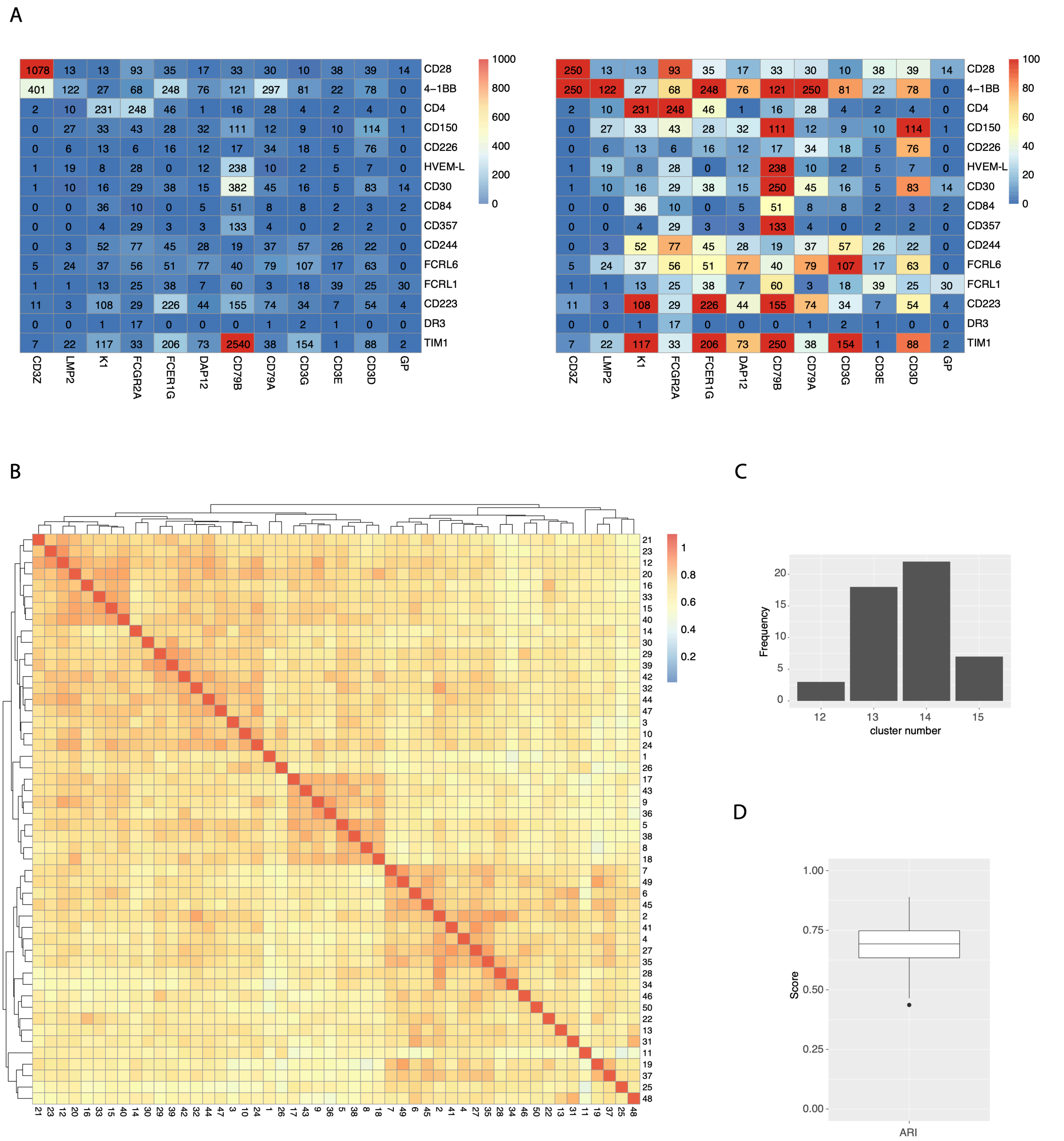

Supplementary Figure 4: scRNA-seq clustering analysis is robust to cell subsampling.

a Heatmaps showing the numbers of cells assigned to each CAR variant before (left) and after (right) subsampling a maximum number of 250 cells from each CAR variant. Of note; CD28-CD3ζ and 4-1BB-CD3ζ T cells were spiked-into the pooled library of CAR T cells prior to co-culture. **b** Matrix of the adjusted Rand index (ARI) of the first 50 seed outputs for the clustering analysis featured in Figure 2 of the main text. This analysis relies on the random subsampling of 250 cells from each CAR variant with larger cell numbers and 500 cells from each negative control sample to balance the dataset. We tested how likely the subsampling can result in aberrant clustering by simulating 50 clustering procedures and measuring the similarity of the top differentially expressed genes using the ARI in a pairwise fashion. **c** The number of clusters identified by the procedure throughout the 50 seeds can vary from 12 to 14. **d** The distribution of ARIs is centered around 0.69 (lower and higher quartile 0.63 and 0.75 respectively, maxima 0.89 and minima 0.43). n=225 independent comparisons.

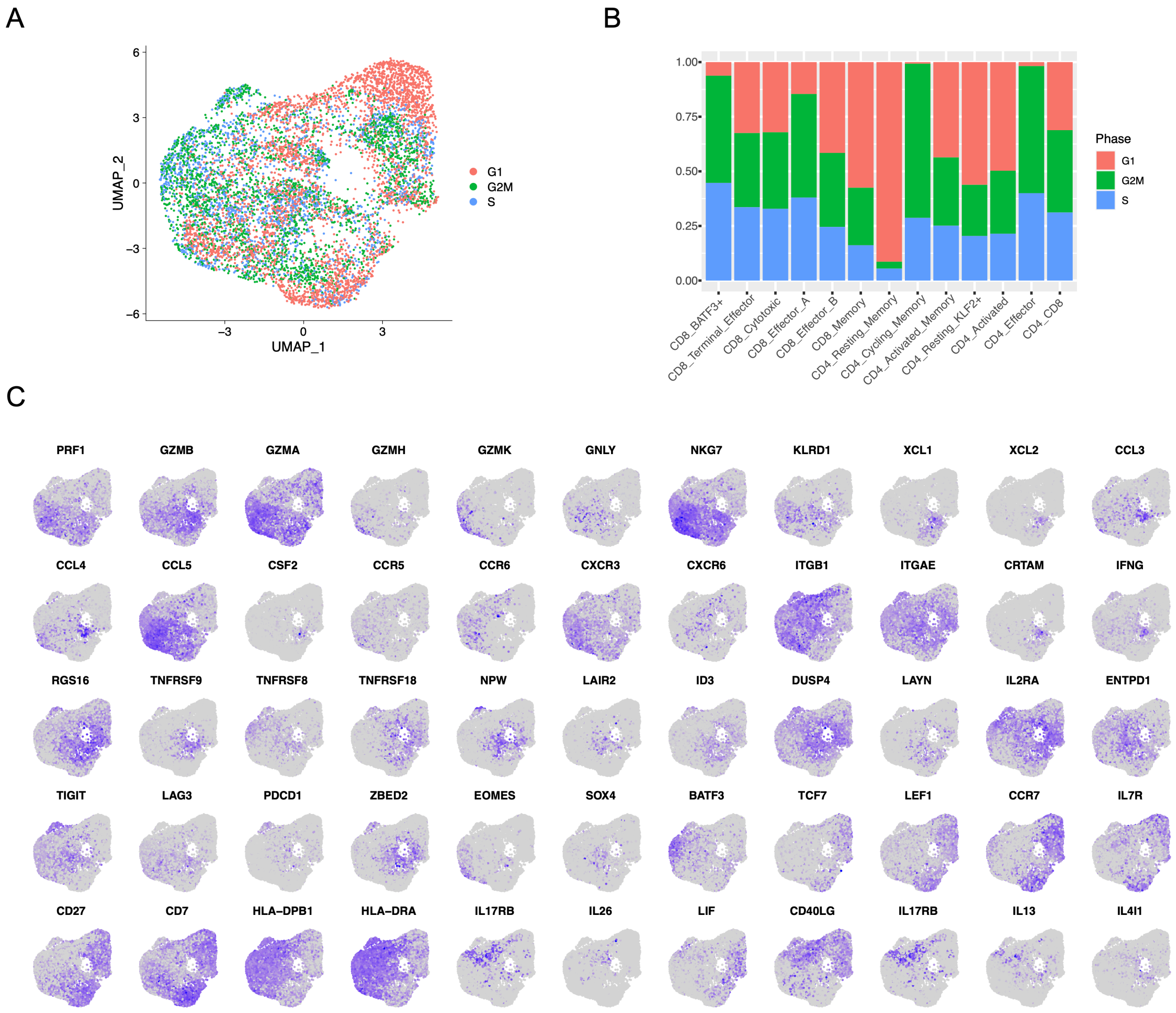

Supplementary Figure 5: Single cell sequencing analysis of pooled library CAR T cells following tumor cell co-culture.

a Cell cycle phase prediction overlaid on its UMAP embedding. **b** Cell cycle phase enrichment across the different clusters annotated in Figure 2. **c** Feature plots showing the distribution of expression of a selection of genes across the UMAP embedding.

A

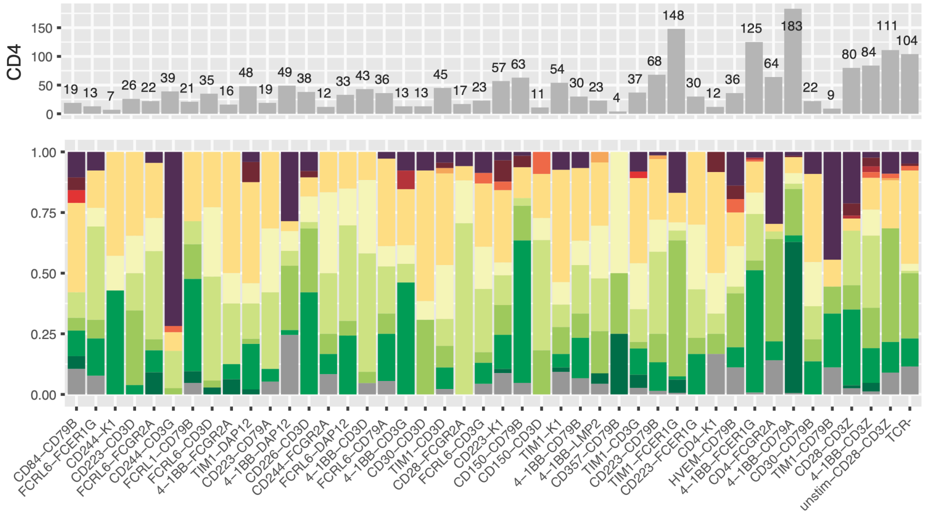

B

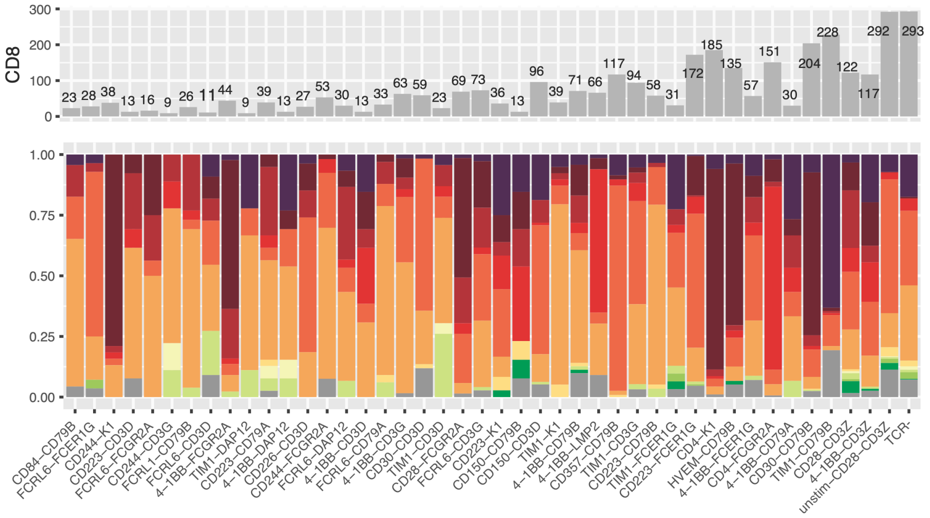

Supplementary Figure 6: CD4 and CD8 cluster enrichment.

Cluster enrichment of CD4 (**a**) or CD8 cells (**b**) observed for the top 40 most represented CAR variants, benchmark controls (CD28-CD3Z and 4-1BB-CD3Z) and negative controls (hT and unstim-CD28-CD3Z). The top panel displays the total counts of CD4 or CD8 cells for each sample group and the bottom panel the fraction of cells found in each of the clusters in Figure 2.

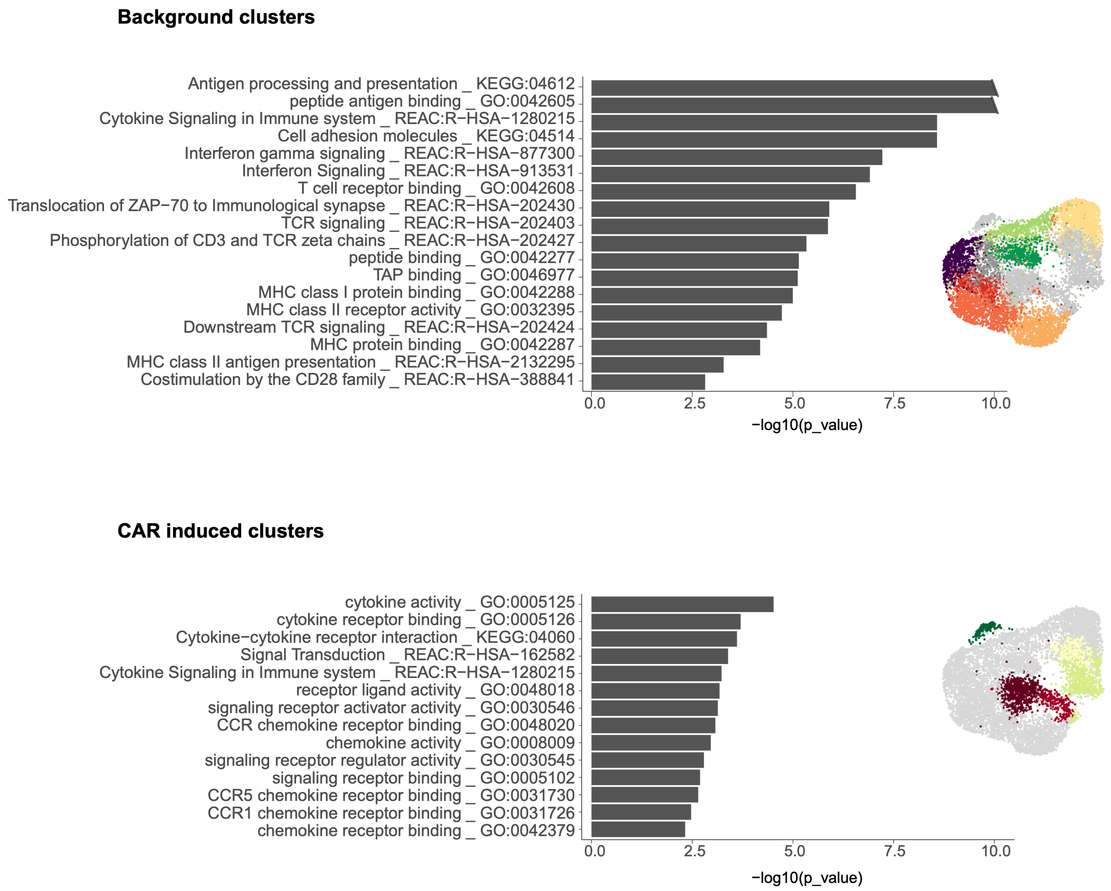

Supplementary Figure 7: Pathway enrichment analysis of CICs and nonCICs.

Pathway enrichment analysis on the differentially expressed genes between CAR induced clusters and background clusters defined in Figure 2. For each group, a selection of the most immunologically relevant gene sets is shown. On the right UMAP plots are shown highlighting the clusters that integrate each group. Statistical significance was determined using the adjusted p-values generated using g:SCS method from g:Profiler for multiple comparison testing.

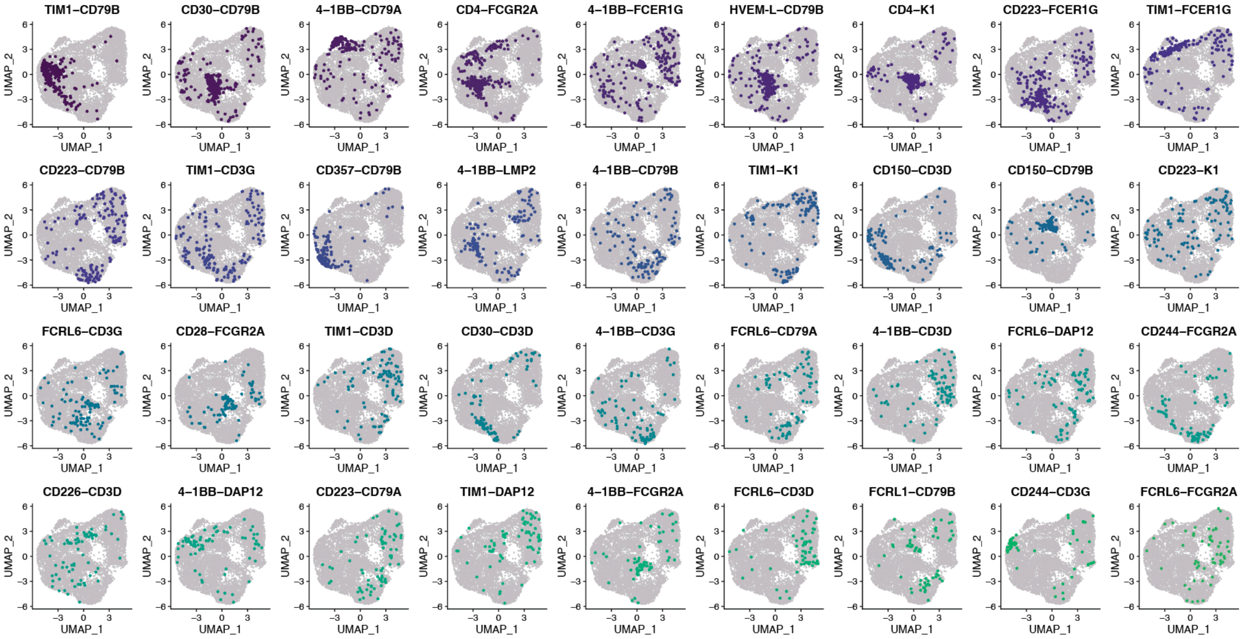

Supplementary Figure 8: After demultiplexing CAR variants map on to different regions of the T cell phenotypic landscape.

Distribution of cells for the top 36 CAR variants within the UMAP embedding of Figure 2.

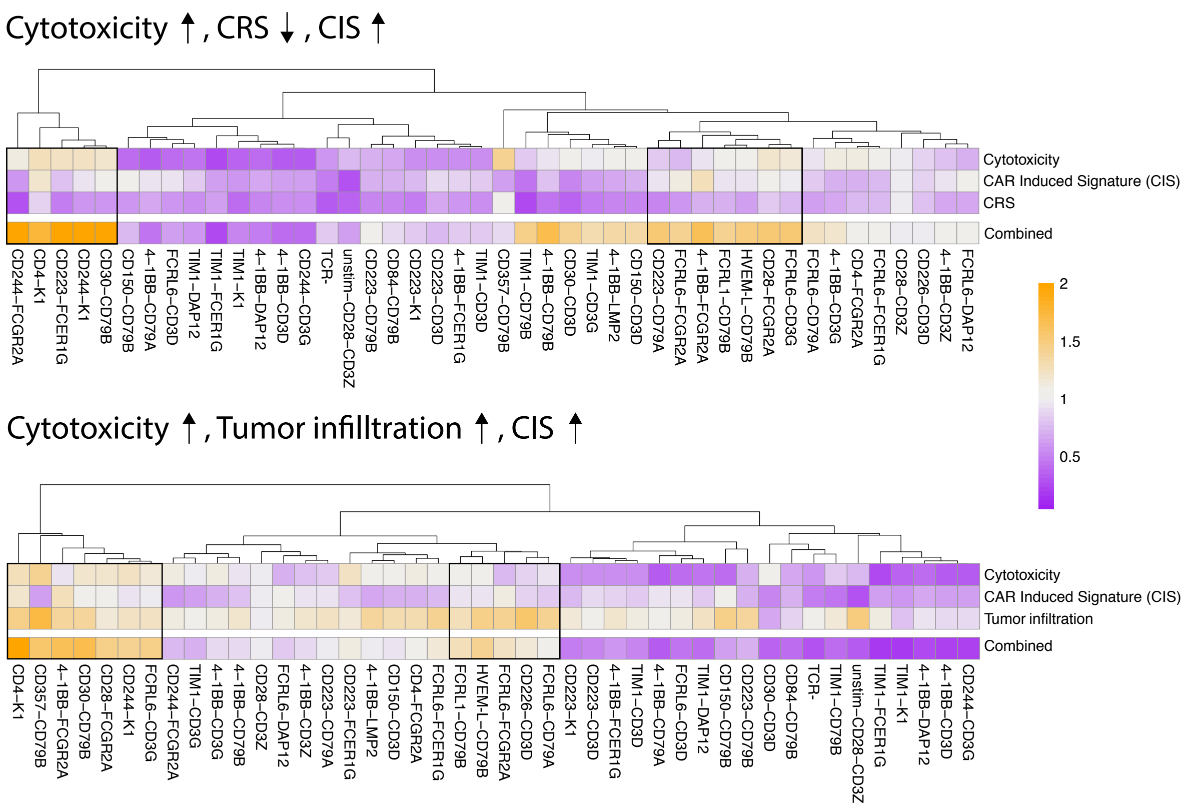

Supplementary Figure 9: Phenotypic characterization of CAR variants based on the combination of gene set expression scoring.

Heatmap showing the difference in gene set scores between the 40 most represented CAR variants, stimulated and unstimulated 28z CAR, BBz CAR and TCR- T cells, for gene sets of choice. The mean score per variant is given as a fold change measurement when compared to 28z WT CAR. The mean score fold change of the different gene sets is later on combined by multiplying or dividing the fold change values according to whether the gene sets are aimed to be enriched or depleted.

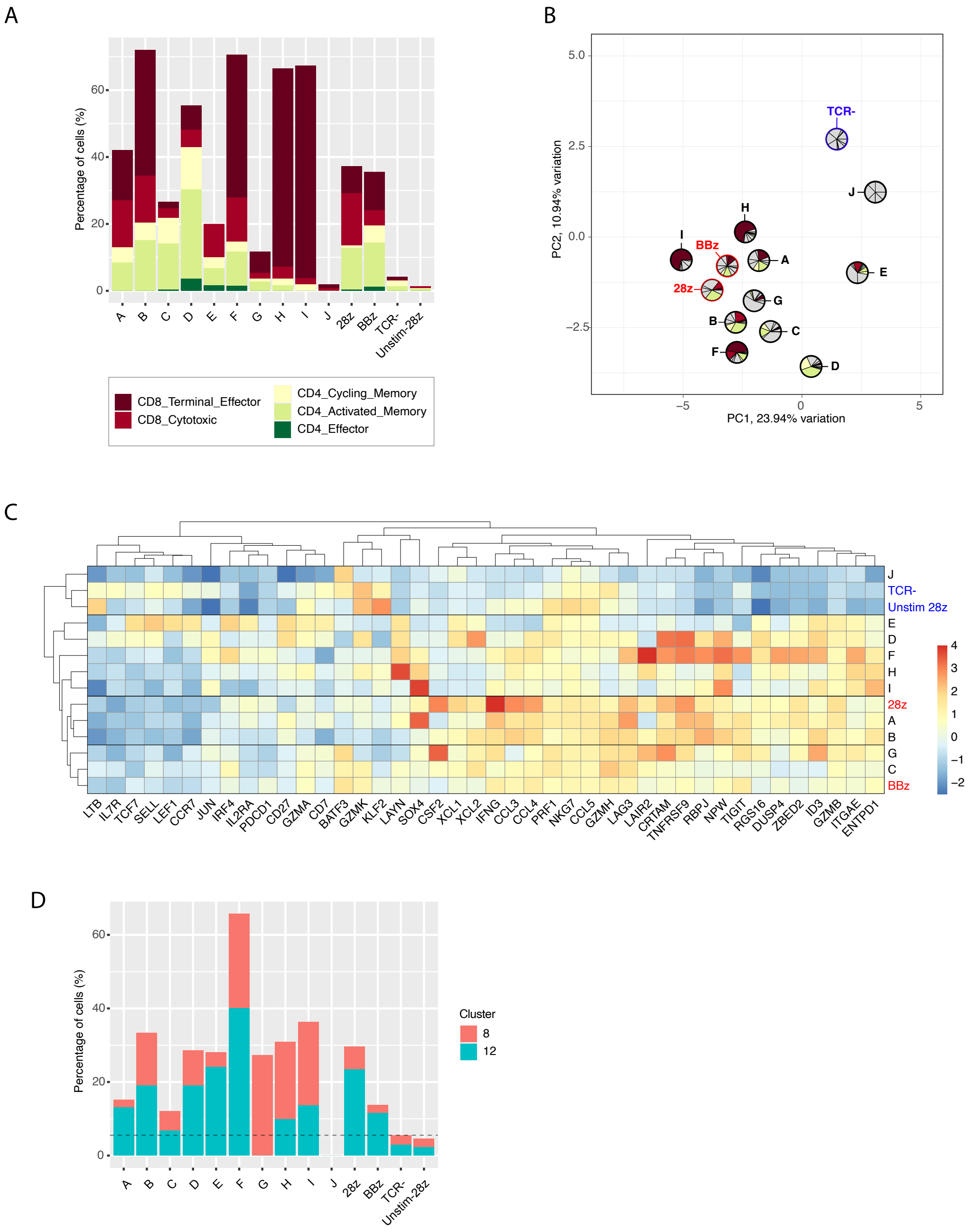

Supplementary Figure 10: Transcriptome guided selection of functional CARs.

a Bar plot depicting the percentage of cells per CAR variant that belong to the 5 different CICs described in Figure 2. **b** Principal component analysis (PCA) of pseudo-bulked scRNA-seq data of a selection of CAR candidates, 28z and BBz CARs coloured in red and TCR- T cells coloured in blue. To avoid batch effect variation only data from Donor 3 is used. Overlayed over each data point, pie charts represent the enrichment of cells in CICs from Fig. 2f. **c** Expression levels of a set of 42 T cell marker genes across CD8+ pseudo-bulked scRNA-seq samples of a selection of CAR variants, 28z and BBz benchmark CAR T cells, TCR- T cells and unstimulated 28z CAR T cells. **d** Enrichment of cells in tumor reactive TIL associated clusters 8 and 12, identified in figure 4, across a selection of CAR variants.

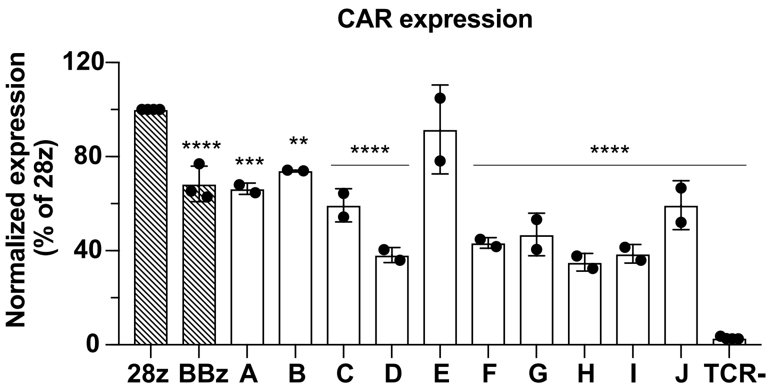

Supplementary Figure 11: The CAR signaling variants are expressed at different levels on the surface of primary T cells than 28z CAR.

Bar plot showing CAR surface expression levels from genome-edited primary T cells, as assessed by flow cytometry and detection of the Strep tag II. To assess significant differences between each variant and 28z, one-way ANOVA and Dunnett's multiple comparisons test was used with the following significance indicators: * p-value < 0.05, ** p-value < 0.01, *** p-value < 0.001 and **** p-value < 0.0001. TCR- refers to T cells without a TCR and error bars represent the S.D. (n=2 independent experimental replicates for variants A-J and n=4 for control groups). Source and statistical data are provided as a Source Data file.

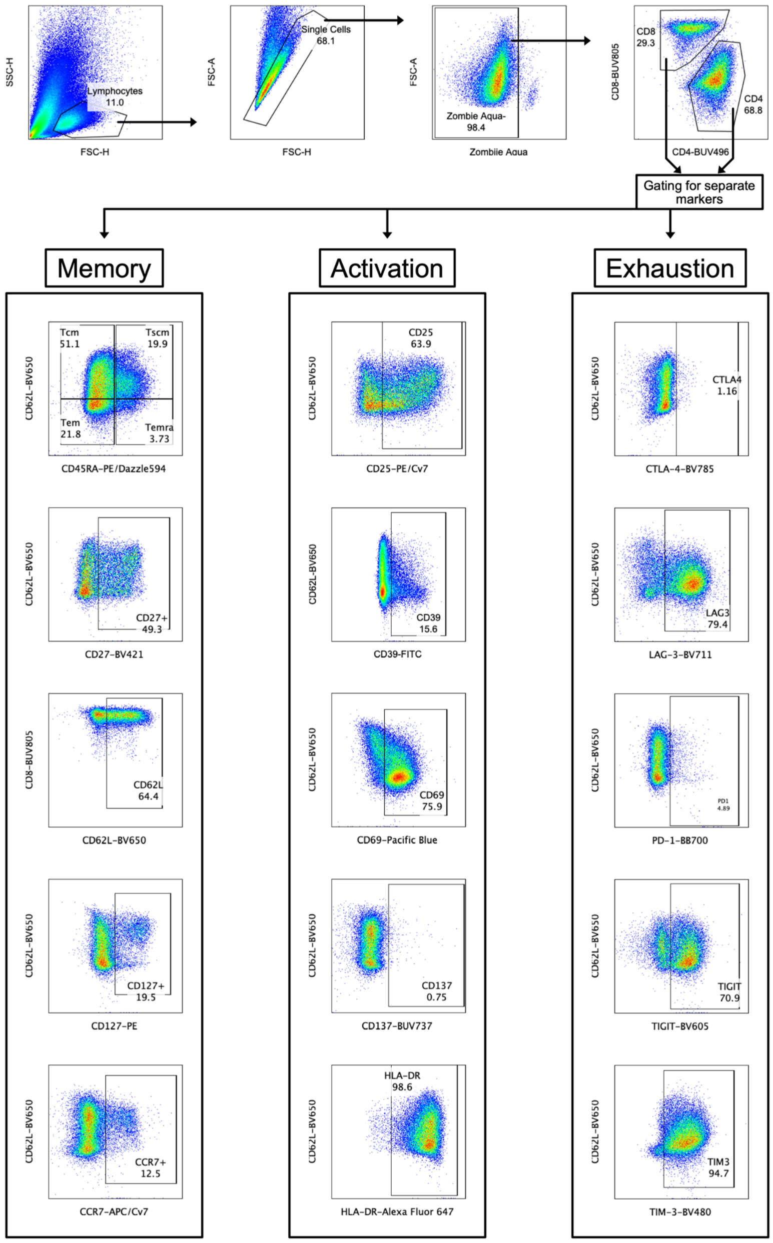

Supplementary Figure 12: Flow cytometry gating strategy used to determine the expression of a panel of T cell surface markers.

A

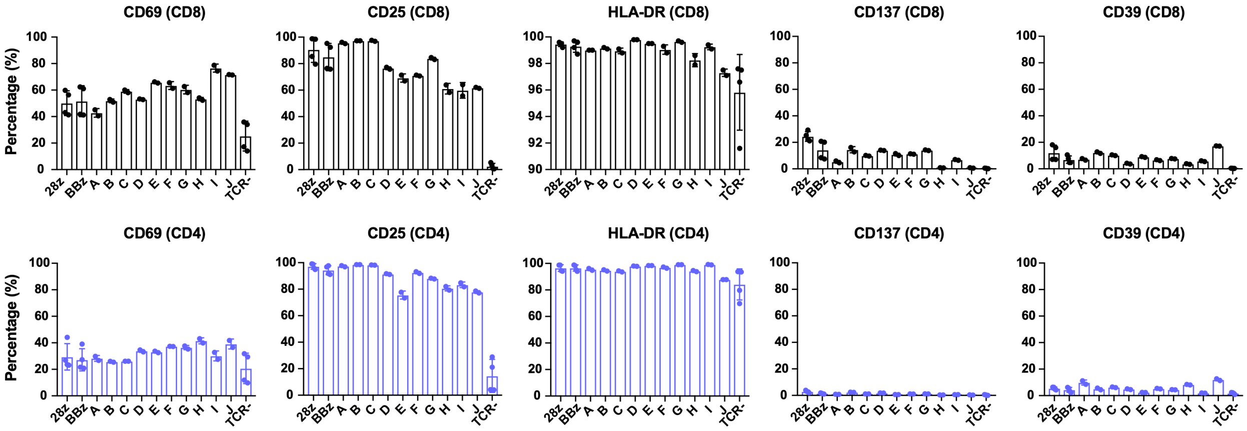

B

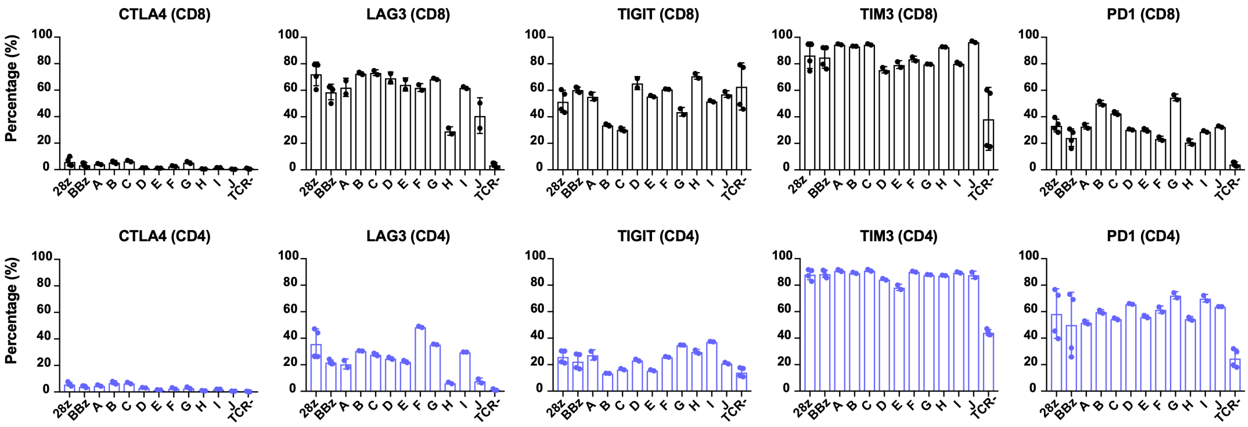

Supplementary Figure 13: Comparison of surface marker expression across CAR variants following tumor co-culture. Percentage of T cells expressing individual T cell surface markers across different CAR variants, 28z and BBz benchmark CARs and TCR-negative T cells following a 4 day co-culture with SKBR3 cells (4:1 E:T ratio). A panel of different T cell activation markers (**a**) and T cell exhaustion markers (**b**) was chosen and measured by flow cytometry. In all panels, TCR- refers to T cells without a TCR and error bars represent the S.D. (n=2 independent technical replicates for variants A-J and n=2 technical replicates from 2 independent experiments for control groups). Source data are provided as a Source Data file.

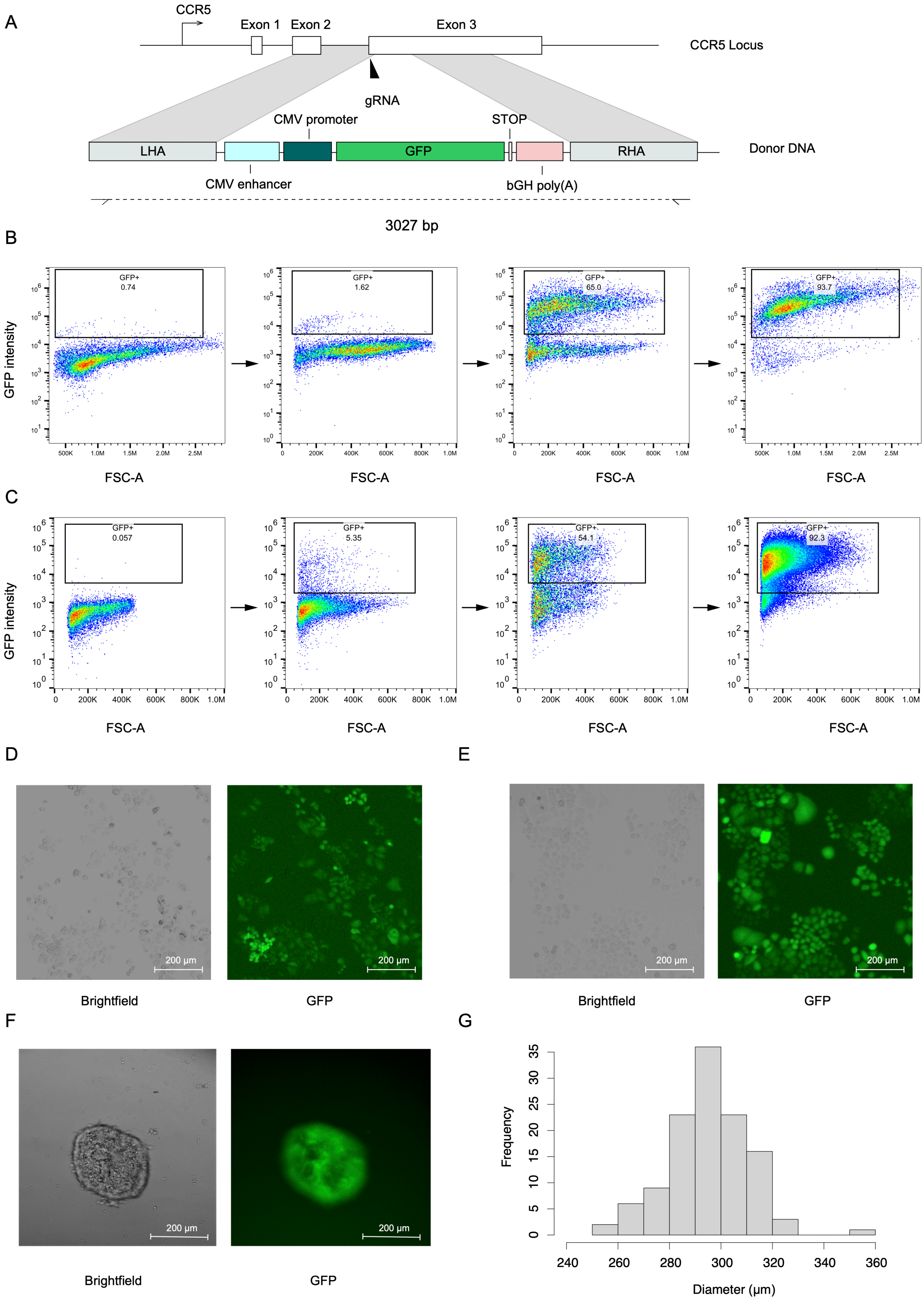

A
CCR5
Exon 1
Exon 2
Exon 3
CCR5 Locus
gRNA
CMV promoter
STOP
LHA
GFP
RHA
Donor DNA
CMV enhancer
bGH poly(A)
3027 bp
B
GFP intensity
GFP+ 0.74
GFP+ 1.62
GFP+ 65.0
GFP+ 93.7
FSC-A
C
GFP intensity
GFP+ 0.057
GFP+ 5.35
GFP+ 54.1
GFP+ 92.3
FSC-A
D
200 μm
Brightfield
GFP
E
200 μm
Brightfield
GFP
F
200 μm
Brightfield
GFP
G
Frequency
Diameter (μm)

Supplementary Figure 14: Genomic engineering of the cancer cell lines for imaging-based cytotoxicity assays.

a The CRISPR-Cas9-based strategy for integrating a GFP expression cassette in the genome of the cell lines SKBR3 and MCF-7. A gene expression cassette was constructed harboring the two-part cytomegalovirus (CMV) promoter, the GFP ORF and a polyadenylation signal. This cassette was flanked by DNA regions homologous to the *CCR5* genomic locus and amplified by PCR to generate the repair template for HDR. A guide RNA targeting the beginning of the third exon of *CCR5* was used to generate Cas9 RNP and transfected in target cells alongside the repair template. **b and c** The transfected SKBR3 and MCF-7 cells respectively were sorted by FACS iteratively to select GFP-expressing cells and obtain a mostly pure (>90%) population. **d and e** Fluorescence microscopy confirmed the clear visibility of SKBR3 and MCF-7 cells respectively. **f** Fluorescence image of MCF-7-GFP cells forming a spheroid structure. **g** Frequency distribution histogram of the diameters in microns of 120 MCF-7 spheroids three days after seeding (the start of live imaging experiments).

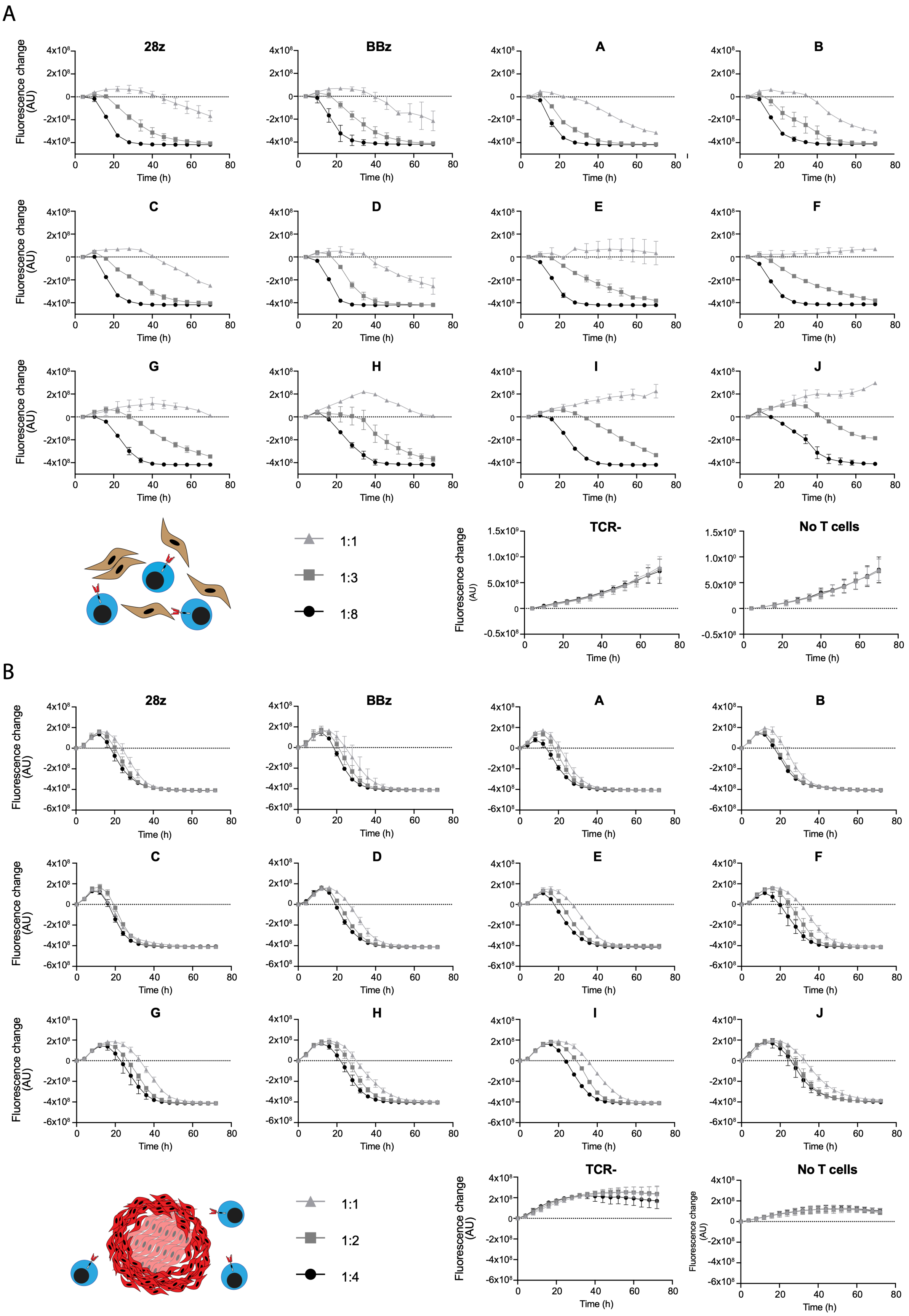
A
28z
BBz
A
B
C
D
E
F
G
H
I
J
Fluorescence change (AU)
Time (h)
1:1
1:3
1:8
TCR-
No T cells
B
28z
BBz
A
B
C
D
E
F
G
H
I
J
Fluorescence change (AU)
Time (h)
1:1
1:2
1:4
TCR-
No T cells

Supplementary Figure 15: The CAR T cell-mediated cytotoxicity of target cells proceeds at different rates depending on the signaling domain combinations used.

CAR T cell-mediated cytotoxicity of HER2+/GFP+ tumor cells quantified over time by fluorescence microscopy. The curves represent the difference in GFP intensity with regards to time point 0. CAR T cells were co-cultured at different E:T ratios with either SKBR3 adherent cells in a "sparse" 2D culture in **a** or with a single tumor spheroid of MCF-7 cells in **b**. Colours indicate different E:T ratios. In all panels, TCR- refers to T cells without a TCR and error bars represent the S.D. (n=2 independent technical replicates for variants A-J and n=2 technical replicates from 2 independent experiments for control groups). Source data are provided as a Source Data file.

Supplementary Table 1: crRNA sequences used in this study

**Genomic target**	**crRNA sequence (5’ to 3’)**
TRAC	CAGGGUUCUGGAUAUCUGU
CCR5	UGACAUCAAUUAUUAUACAU

Supplementary Table 2: Oligonucleotides used in this study

Name	Sequence (5' to 3')	Purpose
F1	gttacaggCACCTGCaacaGGTG	To amplify domains from pool A for cloning
R1	ggaactccCACCTGCcttgTGCTga	
F2	ttagcccaCACCTGCgggcAGCA	To amplify domains from pool B for cloning
R2	ccgagggcCACCTGCtcatGCGG	
F3	CGGGACTAGTGGCgtcGGTTCTGGATATCTGTGGGCTGCCAGAGTTATATTGCTGGGGTT	To amplify HDR repair template with tCTS for CRISPR-Cas9 genome editing.
R3	CACTTCCAGCACCgtcGGTTCTGGATATCTGTGGGCGAGACCACCAATCAGAGGAGTTT	
F4	GCTTGCTAGTAACAGTGGCCTTTAT	To amplify the recombined A and B domains within the CAR gene for sequencing
R4	TACAGGCCTTCCTGAGGGTTCTT	
F5	GGTCAGACAAGCTCCCGGAAAAGGA	To amplify the cytoplasmic region of the CAR gene in the *TRAC*locus for sequencing
R5	AGGTGTCCCTTCCCTGCTT	
F6	AATGATACGGCGACCACCGAGATCTACACTCTTTCCCTACACGACGCTC	scCAR-seq PCR1
R6	GTGACTGGAGTTCAGACGTGTGCTCTTCCGATCTCACACCCTCAGTTCGAAAAGAGTGC	
F7	AATGATACGGCGACCACCGAGATCT	scCAR-seq PCR2
i7-Read2	CAAGCAGAAGACGGCATACGAGATNNNNNNNNGTGACTGGAGTTCAGACGTGTGCTCTTCCGATC	

Supplementary Table 3: Dilutions and catalogue numbers of used antibodies

**Target**	**Fluorochrome**	**Clone**	**Dilution**	**Source**	**Cat. Nr**
Viability	Zombie Aqua	-	1/1000	Biolegend	423102
CD3	BUV395	UCHT1	1/500	BD Biosciences	563546
CD3	APC	UCHT1	1/200	BD Biosciences	300458
CD4	BUV496	SK3	1/500	BD Biosciences	612936
CD8	BUV805	SK1	1/500	BD Biosciences	612889
CD25	PE/Cy7	M-A251	1/50	BioLegend	356108
CD27	Brillant Violet 421	M-T271	1/33	BioLegend	356418
CD39	FITC	A1	1/50	BioLegend	328206
CD45RA	PE/Dazzle 594	HI100	1/50	Biolegend	304146
CD62L	BV650	DREG-56	1/33	BioLegend	304832
CD69	Pacific Blue	FN50	1/50	BioLegend	310920
CD127	PE	A019D5	1/33	BioLegend	351304
CD137	BUV737	4B4-1	1/250	BD Biosciences	741861
CD152 (CTLA-4)	Brillant Violet 785	BNI3	1/50	Biolegend	369624
CD197 (CCR7)	APC/Cyanine7	G043H7	1/50	BioLegend	353212
CD223 (LAG-3)	BV711	11C3C65	1/50	BioLegend	369320
CD279 (PD-1)	BB700	EH12.1	1/250	BD Biosciences	566460
CD366 (TIM-3)	BV480	7D3	1/250	BD Biosciences	746771
HLA-DR	Alexa Fluor 647	L243	1/50	BioLegend	307622
TIGIT	Brillant Violet 605	A15153G	1/50	BioLegend	372712
StrepTag	Biotin	5A9F9	1/400	GenScript	A01737
SAv	BV421	-	1/200	BD Biosciences	405225
HER2	APC	24D2	1/200	BioLegend	324408
