# Supplementary material for: speedingCARs: accelerating the engineering of CAR T cells by signaling domain shuffling and single-cell sequencing
Source: Nat Commun. 2022 Nov 2;13:6555. doi: 10.1038/s41467-022-34141-8 (PMC9630321; doi:10.1038/s41467-022-34141-8)
Supplement: Supplementary file 3 — Description of Additional Supplementary Files [file 41467_2022_34141_MOESM3_ESM.pdf]

Description of Additional Supplementary Files

Supplementary Data 1: List of gene sets used to do gene set scoring analysis.
