# Supplementary material for: speedingCARs: accelerating the engineering of CAR T cells by signaling domain shuffling and single-cell sequencing
Source: Nat Commun. 2022 Nov 2;13:6555. doi: 10.1038/s41467-022-34141-8 (PMC9630321; doi:10.1038/s41467-022-34141-8)
Supplement: Supplementary file 5 — Reporting Summary [file 41467_2022_34141_MOESM5_ESM.pdf]

nature research

Corresponding author(s): Sai T. Reddy

Last updated by author(s): Sep 28, 2022

Reporting Summary

Nature Research wishes to improve the reproducibility of the work that we publish. This form provides structure for consistency and transparency in reporting. For further information on Nature Research policies, see our Editorial Policies and the Editorial Policy Checklist.

Statistics

For all statistical analyses, confirm that the following items are present in the figure legend, table legend, main text, or Methods section.

n/a	Confirmed	
☐	☒	The exact sample size (*n*) for each experimental group/condition, given as a discrete number and unit of measurement
☐	☒	A statement on whether measurements were taken from distinct samples or whether the same sample was measured repeatedly
☐	☒	The statistical test(s) used AND whether they are one- or two-sided<br>*Only common tests should be described solely by name; describe more complex techniques in the Methods section.*
☐	☒	A description of all covariates tested
☐	☒	A description of any assumptions or corrections, such as tests of normality and adjustment for multiple comparisons
☐	☒	A full description of the statistical parameters including central tendency (e.g. means) or other basic estimates (e.g. regression coefficient) AND variation (e.g. standard deviation) or associated estimates of uncertainty (e.g. confidence intervals)
☐	☒	For null hypothesis testing, the test statistic (e.g. *F*, *t*, *r*) with confidence intervals, effect sizes, degrees of freedom and *P* value noted<br>*Give P values as exact values whenever suitable.*
☒	☐	For Bayesian analysis, information on the choice of priors and Markov chain Monte Carlo settings
☒	☐	For hierarchical and complex designs, identification of the appropriate level for tests and full reporting of outcomes
☒	☐	Estimates of effect sizes (e.g. Cohen's *d*, Pearson's *r*), indicating how they were calculated

Our web collection on statistics for biologists contains articles on many of the points above.

Software and code

Policy information about availability of computer code

Data collection	Microscopy: NIS-Elements
Data analysis	Amplicon sequencing data was analysed using Biostrings package in R (version 2.56.0). scRNAseq data was aligned to the human reference genome using CellRanger (version 3.1.0) and the resulting count matrix was analysed in R using Seurat (version 4.0.1), Ucell (version 1.0.0) and gProfiler (version 0.2.0). For TIL mapping Harmony (version 1.0) was used in combination with Seurat (version 4.0.1). Flow-cytometry data were analysed using FLowJo 10 and microscopy data was analyzed using Fiji (version 2.3.0/1.53q) and Ilastik (version beta 1.4.27).

For manuscripts utilizing custom algorithms or software that are central to the research but not yet described in published literature, software must be made available to editors and reviewers. We strongly encourage code deposition in a community repository (e.g. GitHub). See the Nature Research guidelines for submitting code & software for further information.

Data

Policy information about availability of data

All manuscripts must include a data availability statement. This statement should provide the following information, where applicable:

- Accession codes, unique identifiers, or web links for publicly available datasets
- A list of figures that have associated raw data
- A description of any restrictions on data availability

The scRNA-seq and sc-CARseq data have been deposited in the Gene Expression Omnibus under accession number GSE214231. Genome Reference GRCh38 was used in this study. All data generated or analysed during the study are included in the paper or its supplementary information.

Field-specific reporting

Please select the one below that is the best fit for your research. If you are not sure, read the appropriate sections before making your selection.

- [x] Life sciences
- [ ] Behavioural & social sciences
- [ ] Ecological, evolutionary & environmental sciences

For a reference copy of the document with all sections, see nature.com/documents/nr-reporting-summary-flat.pdf

Life sciences study design

All studies must disclose on these points even when the disclosure is negative.

Sample size	No statistical method was used to predetermine sample size. During sc_RNAseq, data was generated from 3 different donors and only CAR variants with at least 50 assigned cells were taken into consideration. n > 50 was considered enough to draw solid conclusions on our data. experimental duplicates were preformed for every experimental condition. In order to compare data from different independent experiments positive and negative controls (in replicates) were used.
Data exclusions	In few ocasions, outliers resulting from technical problems were excluded from the analysis
Replication	During in vitro functional characterization, technical replicates were performed for all experiments and conditions. Only control groups (two positive and two negative controls) were replicated in independent experiments and replication was successful. Such controls were then used to compare data from different independent experiments.
Randomization	Randomization was used to obtain a balanced representation of CAR variants when performing cell clustering using scRNAseq data. A maximum of 250 cell per variant were randomly sub-sampled for downstream analysis.
Blinding	Blinding was not relevant as data was quantified by software and not subject to investigators input.

Reporting for specific materials, systems and methods

We require information from authors about some types of materials, experimental systems and methods used in many studies. Here, indicate whether each material, system or method listed is relevant to your study. If you are not sure if a list item applies to your research, read the appropriate section before selecting a response.

Materials & experimental systems

n/a	Involved in the study	
[ ]	[x]	Antibodies
[ ]	[x]	Eukaryotic cell lines
[x]	[ ]	Palaeontology and archaeology
[x]	[ ]	Animals and other organisms
[x]	[ ]	Human research participants
[x]	[ ]	Clinical data
[x]	[ ]	Dual use research of concern

Methods

n/a	Involved in the study	
[x]	[ ]	ChIP-seq
[ ]	[x]	Flow cytometry
[x]	[ ]	MRI-based neuroimaging

Antibodies

Antibodies used

HLA-DR-Alexa Fluor 647 (L243) from Biolegend; 1:50 :Cat N: 307622
CD69-Pacific Blue (FN50) from Biolegend; 1:50 :Cat N: 310920
CD25-PE/Cy7 (M- A251) from Biolegend; 1:50 :Cat N: 356108
CD137/4-1BB-PE/Dazzle 594 (4B4-1) from Biolegend; 1:250 :Cat N: 741861
CD45RA-PE/Dazzle 594 (HI100) from Biolegend; 1:50 :Cat N: 304146
CCR7-APC/Cy7 (3D12) from Biolegend; 1:50 :Cat N: 353212
CD27-BV570 (O323) from Biolegend; 1:33 :Cat N: 356418
CD39-FITC (A1) from Biolegend; 1:50 :Cat N: 328206
CD127-PE (A019D5) from Biolegend; 1:33 :Cat N: 351304
CTLA-4-BV785 (L3D10) from Biolegend; 1:50 :Cat N: 369624
LAG-3-BV711 (11C3C65) from Biolegend; 1:50 :Cat N: 369320
TIGIT-BV421 (A15153G) from Biolegend; 1:50 :Cat N: 372712
CD62L-BV650 (DREG-56) from BD Biosciences; 1:33 :Cat N: 304832
CD3ε-APC (UCHT1)from Biolegend ; 1:20 :Cat N: 300458
SAv-BV421 from Biolegend; 1:400 :Cat N: 405225
HER2-APC (24D2) from Biolegend; 1:200 :Cat N: 324408
CD3-BUV395 (UCHT1) from BD Biosciences; 1:500 :Cat N: 563546
CD4-BUV496 (SK3) from BD Biosciences; 1:500 :Cat N: 612936

	CD8-BUV805 (SK1) from BD Biosciences; 1:500 :Cat N: 612889<br>PD-1-BB700 (EH12.1) from BD Biosciences; 1:250 :Cat N: 566460<br>TIM3-BV480 (7D3) from BD Biosciences; 1:250 :Cat N: 746771<br>anti-StrepTag -biotin (5A9F9) from GenScript 1:200 :Cat N: A01737
Validation	The used antibodies are well characterized clones with reported QC by the vendor. Dilutions were further validated using human PBMCs.

Eukaryotic cell lines

Policy information about cell lines

Cell line source(s)	SKBR3 and MCF7 were generously provided by Roger R. Beerli (NBE-Therapeutics) and Madhuri Manivannan (University of Basel) respectively. Original commercial source cannot be determined but cells were authenticated as described below.
Authentication	Cell lines were authenticated by checking the levels of HER2 expression by Flow Cytometry
Mycoplasma contamination	Cell lines were not tested for micoplasma contamination but were cultured with normocin, an anti-mycoplasma antibiotic.
Commonly misidentified lines<br>(See ICLAC register)	No commonly misidentified lines were used in the study

Flow Cytometry

Plots

Confirm that:

- [x] The axis labels state the marker and fluorochrome used (e.g. CD4-FITC).
- [x] The axis scales are clearly visible. Include numbers along axes only for bottom left plot of group (a 'group' is an analysis of identical markers).
- [x] All plots are contour plots with outliers or pseudocolor plots.
- [x] A numerical value for number of cells or percentage (with statistics) is provided.

Methodology

Sample preparation	For flow cytometry and cell sorting staining cells were washed in (PBS 2% FBS) and incubated 30 minutes at 4C in FACs buffer (PBS 2% FBS) containing the desired antibodies.
Instrument	Cell sorting was carried out using an SH800 cell sorter (Sony) and flow cytometric analysis was carried out using an Cytek Aurora Flow Cytometry System from Cytek Biosciences.
Software	FlowJo 10 software (BD Biosciences)
Cell population abundance	Flow cytometry was employed to ensure that the purity of a target cell populations was at least two-third of the overall population
Gating strategy	FSC/SSC gating was used to define an initial lymphocyte gate. Positive and negative populations were then quantified after establishing negative gates using unstained controls. For cell sorting an additional singlet gate was incorporated into the gating strategy.

- [x] Tick this box to confirm that a figure exemplifying the gating strategy is provided in the Supplementary Information.
